# Unsupervised Learning and Multipartite Network Models: A Promising Approach for Understanding Traditional Medicine

**DOI:** 10.3389/fphar.2020.01319

**Published:** 2020-08-26

**Authors:** Mohieddin Jafari, Yinyin Wang, Ali Amiryousefi, Jing Tang

**Affiliations:** Research Program in Systems Oncology, Faculty of Medicine, University of Helsinki, Helsinki, Finland

**Keywords:** unsupervided learning, network modelling, precision medicine, traditional medicine, systems medicine

## Abstract

The ultimate goal of precision medicine is to determine right treatment for right patients based on precise diagnosis. To achieve this goal, correct stratification of patients using molecular features and clinical phenotypes is crucial. During the long history of medical science, our understanding on disease classification has been improved greatly by chemistry and molecular biology. Nowadays, we gain access to large scale patient-derived data by high-throughput technologies, generating a greater need for data science including unsupervised learning and network modeling. Unsupervised learning methods such as clustering could be a better solution to stratify patients when there is a lack of predefined classifiers. In network modularity analysis, clustering methods can be also applied to elucidate the complex structure of biological and disease networks at the systems level. In this review, we went over the main points of clustering analysis and network modeling, particularly in the context of Traditional Chinese medicine (TCM). We showed that this approach can provide novel insights on the rationale of classification for TCM herbs. In a case study, using a modularity analysis of multipartite networks, we illustrated that the TCM classifications are associated with the chemical properties of the herb ingredients. We concluded that multipartite network modeling may become a suitable data integration tool for understanding the mechanisms of actions of traditional medicine.

## Introduction

**Figure 1:**
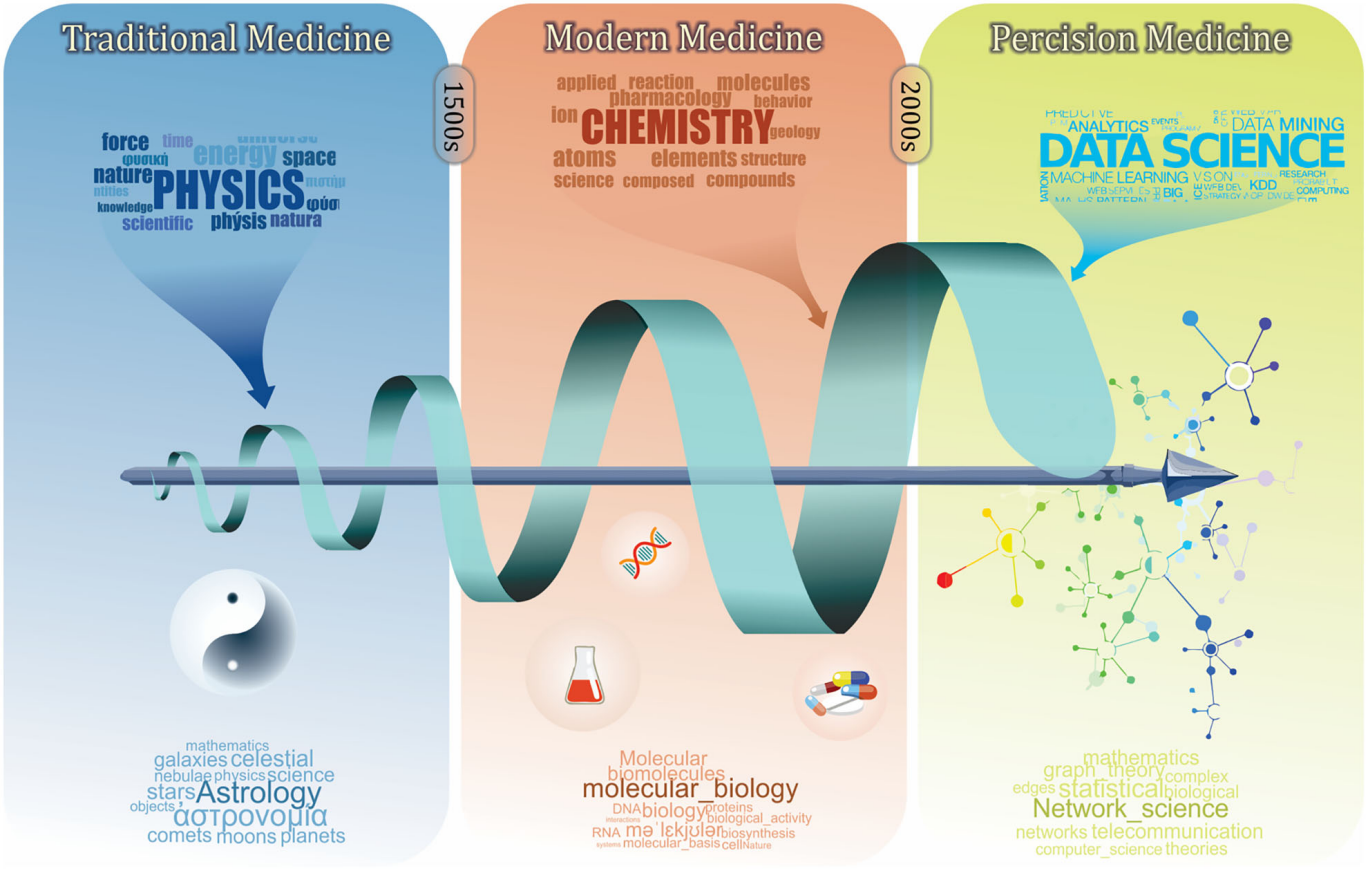
A brief history of medicine and its relation with other branches of science. Traditional medicine as the first era of medicine was mainly built on the physical characterization of diseases and patient biographical data. The modern medicine was established by including more chemical and physical characterizations. Defining biomolecule using biochemistry and molecular biology revealed more details of diseases and pathological processes. This eventually led to the development of diagnosis codes and the pharmaceutical industry. Recently, precision medicine has emerged with the advances of data science, which involves more holistic analyses in order to understand human medicine at the systems level.

Classification and clustering are our fundamental learning process to understand human biology and diseases. To achieve the ultimate goal of precision medicine, i.e., the right intervention for a patient at the right time (Stefano and Kream, 2015), there has been a long history of symptom-based diagnosis that utilizes available information to classify patients, diseases, and drugs (**Figure 1**). In the early days of traditional medicine, physicians tried to characterize diseases using empirical terms, such as temperament and meridian (Rezadoost et al., 2016; Arji et al., 2019; Wang Y. et al., 2019), based on which they prescribed corresponding herbs that are known to target them (Xu, 2011; Li and Weng, 2017). With increasing knowledge on biochemistry, the era of modern medicine has started, further advancing our understanding of human diseases to the molecular level. Molecular profiles, along with clinical phenotypes, are leveraged to formally characterize diseases, disorders, and symptoms (Steindel, 2010).

One of the greatest challenges in precision medicine is to integrate all available patient-derived data for accurate diagnosis and treatment, which would require novel data-driven approaches rather than more conventional hypothesis-driven approaches (Rouillard et al., 2015). Genomic information for patients, albeit fundamental and often necessary, may not be sufficient due to the fact that the human genome is dynamically adjusting its functions by epigenetic regulations (Jafari et al., 2017; Nussinov et al., 2019). Therefore, interactions among other molecular features including transcriptome, proteome, and metabolome should also be considered to obtain a more systematic characterization of the diseases (Eric, 2014). On the other hand, phenotypic data including cell and tissue images have been utilized for illustrating the impact of molecular alternations in human diseases (Langlois et al., 2011; De Fauw et al., 2018). Likewise, to improve our understanding on human diseases, we may also investigate sources of clinical, phenotypic, and pharmacological data that are derived from traditional medicine (Ma et al., 2010; Zhao et al., 2014). A systematic integration of all of these available information may provide a promising approach to turn precision medicine into a reality ultimately.

Here, we started by reviewing the application of clustering analysis in high-throughput biological studies in modern and traditional medicine. Next, we described the application of clustering in network modeling for the stratification of drugs or patients. We focused on the advantages and promises of a particular network modelling approach called multipartite networks which can inherently integrate heterogeneous data types at multiple levels. In a case study, a multipartite network was developed to model traditional medicine herbs. We showed that this modeling approach provides novel insights on the rationale of herb classifications, which may facilitate the drug discovery in TCM, such as discovering herb combinations or prioritization of active ingredients.

## Using Clustering to Improve Patient Stratifications

Thanks to advanced experimental and computational technologies, we are able to collect, standardize, and integrate a variety of cell-based patient-derived datasets. For example, the LINCS program (Keenan et al., 2018) is one of the recent multi-center studies to facilitate the understanding of cancer biology by providing transcriptional and morphological changes of multiple cancer cell lines in responding to a variety of pharmaceutical agents. Moreover, there are national and international efforts to sequence patients’ genomic features. For example, UK Biobank and FinnGen focused on the contributions of genetic predisposition and environmental exposure to the occurrence of common diseases for over half a million subjects (The Finngen Research Project Takes Finns to a Discovery Trip to Genome Data; Manolio, 2018). On the other hand, with the development of computational methods such as text mining technologies, researchers are able to standardize data resources in traditional medicine, making them easily accessible and reusable (Zhou et al., 2005; Mirzaeian et al., 2019). For example, the SymMap database was constructed to provide a mapping relationship between 499 natural products, 19,595 ingredients, 1,717 clinical symptoms, and 5,235 diseases (Wu et al., 2019). This work showed the potential to integrate traditional and modern medicine at both phenotypic and molecular levels toward phenotype-based drug discovery. Another example was the UNaProd database which contains information concerning 3,411 natural products used in Iranian traditional medicine (ITM) (Naghizadeh et al., 2020).

Clustering has been commonly used to identify subpopulations of patients with distinctive genetic variants or gene expression profiles. For example, Naval et al. showed how clustering analysis helped identifying single nucleotide polymorphisms (SNPs) associated with skin properties (Naval et al., 2014). Combining transcriptomic data with images, Voineagu et al. showed how the clustering methods characterize distinct complex disease subtypes in autism spectrum disorder (Voineagu et al., 2011). Additionally, in clinical proteomics, clustering analysis also identifies a group of proteins as functional modules in pathogenesis. For example, Baldelli et al. clustered non-small cell lung cancer tumors according to the expression, activation, and phosphorylation levels of 26 signaling proteins (Baldelli et al., 2015). Implementing clustering methods in the context of precision medicine is not only applicable to omics data, but also to physiological data. For example, Xu et al. developed human stress management using clustering of physiological signals during series of task-rest cycles (Xu et al., 2015). On the other hand, image data as a major part of health records of individuals is commonly utilized (Hsu et al., 2013). For example, Enguehard et al. presented a strategy of integrating neural network and clustering analysis for automatic magnetic resonance imaging data analysis (Enguehard et al., 2019). Furthermore, it has been shown that utilizing biomedical annotations can potentially improve clustering analysis to obtain more biologically relevant disease categories (Futschik and Carlisle, 2005; Bandyopadhyay et al., 2007; Lee, 2011). Therefore, the integration of existing biomedical annotations, such as gene ontology or pathway enrichment, is also expected to improve patient disease clustering with refined distance functions (Handl et al., 2005).

As abovementioned, exploring subclasses of diseases and drugs is a prevalent task in precision medicine, and traditional medicine is no exception. For example, Liu et al. studied the gene expression signature of breast cancer cell lines for an herbal formula Si-Wu-Tang (SWT). This analysis showed that the effect of SWT is comparable to β-estradiol treatment on estrogen-responsive genes (Liu et al., 2013). Ruan et al. proposed a clustering algorithm called THCluster that can effectively discover meaningful categorization of herbs and their potential clinical indications (Ruan et al., 2017). Zhang et al. validated TCM syndrome types using a clustering method based on latent tree models, based on which they proposed a standard for syndrome differentiation in TCM which was then validated successfully in a study of kidney deficiency (Zhang et al., 2008). Likewise, Zhao et al. proposed a top-down subspace clustering for improving the precision of syndrome differentiation. Considering 5,600 symptoms and 150 syndrome elements of AIDS (acquired immune deficiency syndrome) patients, they showed that their method identified clusters of patients more precisely, compared to conventional clustering algorithms such as k-means (Zhao et al., 2014).

While identifying the heterogeneity of patients is critical, understanding the driving molecular mechanisms of such heterogeneity shall provide more rational on the design of precision medicine. To understand the underlying factors that are shared by patients with similar diseases, more information about the interaction of biological entities including genes, proteins, and drugs is required. By introducing network models, such a complex layer of information can be systematically evaluated, for which clustering analysis may further help infer the distinctive disease patterns. In the following we focused on the combination of network modeling and clustering analysis and showed that how they may contribute to the understanding for precision medicine.

## Exploring Network Modules as a Basis for Classification

A simple network consists of a set of elements called nodes or vertices which are connected by a set of links or edges (Jafari et al., 2013). Depending on the definition of node and edge sets, numerous types of biological networks can be constructed and used for further analysis. For example, the degree of a node, which is defined as the number of links attached to the node, suggests the importance of node and helps detect global and provincial hubs within the network. The heterogeneity of a network which is defined as the root of the variance of degrees divided by their mean, also explains the overall topology of the network and organization of relationships among the nodes (Dong and Horvath, 2007).

**Figure 2:**
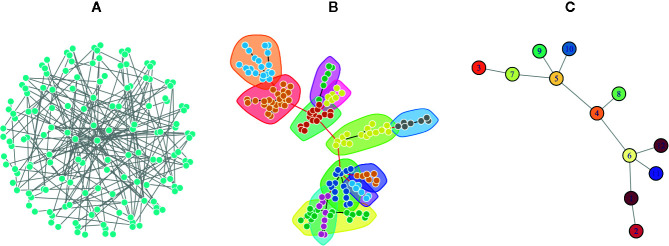
A Poisson-distributed random network is represented in three formats. **(A)** A complex view of the network by placing nodes on a sphere layout that obscure the complexity of the network topology. **(B)** The same network using the force-directed layout algorithm in which communities were identified *via* a greedy optimization of modularity score. This representation is usually used to show the modular structure of the network. **(C)** The simplified network in which each node represents a community in the original network, and each edge denotes the interaction of communities.

One of the major network modeling approaches is the modularity analysis or community detection which is the intersection of clustering analysis and network science (Fortunato, 2010; Fortunato and Hric, 2016). In this analysis, exploring the local densely connected nodes, i.e., networks community structure is the main aim. In other words, a community within a given network includes nodes with high intra-relationship and low inter-relationship with the other nodes outside the community (Girvan and Newman, 2002). Therefore, finding network modules is important to elucidate and understand the complex topology of networks by discriminating dense and sparse local structures. The network topology determines the adjacency matrix, which can be utilized for clustering analysis alone or in combination with the similarity matrix derived from the node properties (Von Luxburg, 2006; Fortunato, 2010). Since in real biological and disease networks, there are multifunctional nodes belonging to more than one group, soft clustering is also recommended (Yang and Leskovec, 2015). In the soft clustering, overlapping communities, also called covers, are detectable because the multiple memberships for a node are allowed. There are other methods including dynamical clustering (Jeub et al., 2015). For example, the Markov Clustering algorithm (MCL) is one of the commonly used dynamic clustering algorithms based on biological annotations (Jafari et al., 2015). After exploring the modules of a given network, it is common to convert the network into its reduced version, where a node of the reduced network corresponds to a module of the original network, and an edge is inferred from the number of interactions between the modules (**Figure 2**).

Utilizing molecular information in conjunction with the module detection to predict biological functions is a common task (Vespignani, 2003; Jafari et al., 2015). The main assumption is that members of the same cluster tend to be involved in the same biological process which is known as guilt-by-association. For example, in protein-protein interaction networks, clique-based clustering was used to detect protein complexes (Altaf-Ul-Amin et al., 2006; Phan and Sternberg, 2012; Jafari et al., 2013). Hierarchical clustering was utilized to identify signaling cascades or metabolic pathways (Guimera and Amaral, 2005; Koch and Ackermann, 2013; Azimzadeh Jamalkandi et al., 2016). Also, according to the local topological features of biological networks, clustering methods are commonly used to predict cellular co-localization and co-expressed gene regulatory mechanisms (Dittrich et al., 2008; Amiri et al., 2013; Mitra et al., 2013). At the phenotypic level, network modeling linking phenotypes to molecular components of a biological system, e.g., disease-causing genetic variations is also one of the exciting research areas (Goh et al., 2007; Loscalzo and Barabasi, 2011; Goh and Choi, 2012; Emmert-Streib et al., 2013). In the context of traditional medicine, Huang et al. highlighted how network pharmacology modeling allows us to integrate concurrent and traditional knowledge of herbal medicines for the development of new drugs for complex human diseases (Huang et al., 2013). Using a network-based integration of chemical structure and omics data, they inferred novel drug-disease interactions *via* molecular targets and pathways. Similarly, Li et al. introduced a distance-based mutual information model to score herb interactions based on their frequencies and distances, and thus identify the rationale of herb combinations (Li et al., 2010).

Network biology approaches have also shown potential for exploring disease subcategories and patient subclasses in TCM. For instance, Zhou et al. constructed a clinical phenotype network to investigate the underlying mechanisms of TCM diagnosis and treatment (Zhou et al., 2014). Wang et al. proposed a co-occurrence network approach to identify the TCM symptoms as biomarkers for the fatty liver disease (Wang W. et al., 2019). Interestingly, Jiang et al. also demonstrated the association between the TCM symptoms and tongue-coating microbiome using co-occurrence networks (Jiang et al., 2012). Network modeling of cold and hot syndromes of traditional medicine has also been developed. For example, Ma et al. provided a gene expression signature of the cold syndrome in TCM associated with the neuroendocrine-immune system. By analyzing the protein interaction networks, they showed that the genes related to the cold syndrome are involved in pathways of energy metabolism, neurotransmitters, hormones, and cytokines (Ma et al., 2010). Likewise, Lu et al. provided distinctive molecular signatures in CD4-positive T cells of Rheumatoid Arthritis patients associated with the cold and heat patterns in TCM respectively (Lu et al., 2012a; Lu et al., 2012b).

## Multipartite Network Models for Integrating Heterogeneous Data

With the development of high-throughput technologies, precision medicine has been made more plausible with increasingly diversified data sets. These data sets range from gene expression profiles to medical images, where the scales, characteristics, and formats are different since they are gathered at the different levels of biological systems (Lee, 2011). The integration of information from these heterogeneous biological and clinical data sets need to be applied in order to discover new mechanistic insights of systems medicine. For example, to predict more effective disease treatment options using multi-targeted drug combinations (Tang and Aittokallio, 2014), we need to gather multiple data types such as *in vitro* drug response of cancer cells and *in vivo* response of patients including symptoms and molecular profiles. To predict the effectiveness of drug combinations, understanding about the signaling pathways and drug target interactions along with the pathophysiological states is essential. There is a major type of network models called multipartite networks which are commonly used in systems medicine (Junker and Schreiber, 2008). This kind of network modeling is crucial due to its flexibility to integrate mixed datasets and discover complex hidden relationships which are required for understanding precision medicine. Unlike ordinary uni-partite networks which contain single sets of nodes and edges represented by an adjacency matrix, a multipartite network constitutes of multiple sets of nodes and edges which are exemplified by incidence matrix (Agnarsson and Greenlaw, 2007). Depending on the data types, the network can represent gene-disease (Bauer-Mehren et al., 2011; Barneh et al., 2016; Chen et al., 2018), drug-target (Barneh et al., 2016), protein-cell localization (Mirzaei Mehrabad et al., 2018), drug-disease (Lamb, 2007) and drug-side effect associations (Luo et al., 2014), as well as associations at the patient level including patient-drug interactions and patient-symptom interactions (Bhavnani et al., 2010).

Based on the constructed multipartite networks, different kinds of clustering algorithms can be applied to identify the hidden subnetwork structures for each node set. For instance, Long et al. proposed a clustering method by a combination of co-clustering and probabilistic hidden Markov models (Long et al., 2007). Also, Hartsperger et al. developed a fuzzy multipartite clustering to decompose the nodes of multiple types in tripartite networks (Hartsperger et al., 2010). They showed that the fuzzy clustering algorithm was able to identify functionally correlated modules of a tripartite gene-disease-protein complex network for the identification of biologically meaningful clusters. Duan et al. identified two major subtypes of breast cancer by reconstructing a tripartite graph of drug-cell line-patient tumors. They showed how drug response data helped discover dysregulated pathways for breast cancer (Duan et al., 2013). A multipartite network can be also utilized as a visualization tool, with which one can navigate efficiently the high-throughput drug response data from public databases including Cancer Cell Line Encyclopedia (CCLE) and Genomics of Drug Sensitivity in Cancer (GDSC) (Duan et al., 2014).

**Figure 3:**
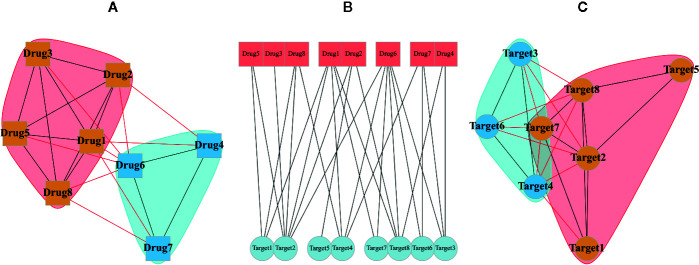
A random bipartite graph or bigraph of drugs (square nodes) and targets (circular nodes) is represented along with two possible projections of it. The center graph **(B)** is a main bipartite graph by placing nodes in two different sets. The projection of drugs **(A)** and the projection of targets **(C)** are shown in which communities were identified *via* greedy optimization of modularity score.

Typical multipartite network analyses involve network projection, which aims for simplifying the network topology from the viewpoint of each node set separately. In **Figure 3**, the projection of a schematic bipartite network and module detection analysis is briefly presented. Constructing the projected networks facilitates the exploration of hidden relationships among each set of nodes in a multipartite network. For example, Barneh et al. constructed a drug-target network and further developed its projected version called Drug Similarity Network (DSN) and Target Similarity Network (TSN), which can be used for drug-target prediction (Barneh et al., 2016). Recently, they have applied the method to predict drug combinations, and confirmed them experimentally (Barneh et al., 2018; Barneh et al., 2019). To facilitate drug repositioning, network topological similarity-based inference (NTSIM) and its classification-equipped version, i.e., NTSIM-C methods were also proposed to unveil novel drug-disease associations (Zhang et al., 2018).

## Network Analysis Rationalizes TCM Classifications: A Case Study

The idea of utilizing multipartite networks in traditional medicine is potentially feasible, as the data standardization and annotation has been increasingly pursued. However, to the best of our knowledge, the models are not yet utilized to provide more profound insights on traditional medicine, although some tools such as SymMap (Wu et al., 2019) may provide an appropriate dataset to build such multipartite networks. In the following, we conducted a case study to reconstruct a bipartite network of natural products and ingredients of TCM to show the potential of this modeling for understanding TCM rationale for disease treatment and drug discovery.

TCM-related databases provide a large set of information about the TCM herbs including their classifications and disease indications, as well as molecular characterization, such as ingredient profiles and molecular targets. Recently, this information has been successfully applied for developing computational models to understand the TCM classifications (Wang Y. et al., 2019). As a case study to show the potential of multipartite networks in the integration of heterogonous data, we obtained a list of 4,485 natural products consisting of 2,857 chemical ingredients from the TCMID database (Huang et al., 2018). We used the second version of the TCMID database, as the largest dataset in this field, which contains richer experimental data originating from ingredient-specific and herbal mass spectrometry spectra. The natural products and ingredients are considered to be the two parts of a bipartite network. After removal of disconnected nodes, we extracted a giant component of the graph consisting of 7,004 nodes and 17,555 edges. Following the projection of this bigraph as outlined in **Figure 3**, two projected graphs called the natural product similarity network (NSN) and ingredient similarity network (ISN) were reconstructed, such that each edge indicates at least one common ingredient or natural product in NSN and ISN, respectively. The NSN contains 4,308 natural products and 204,807 edges, while the ISN consists of 2,696 nodes and 78,228 edges. The community detection was subsequently done for both similarity networks *via* optimizing a modularity score (Clauset et al., 2004), resulting in 42 and 24 communities for NSN and ISN, separately. The fast greedy algorithm outperformed compared to the other high-performance algorithms, i.e., infomap and walktrap (Labatut and Balasque, 2013; Wagenseller et al., 2018) according to the highest average of modularity index in the NSN and ISN (**Supplementary file 2**). These communities reflect the internal similarity of herbs and ingredients which could be investigated further. For example, a community of NSN indicates a set of natural products with similar profiles of ingredients. Therefore, the members of the same natural product cluster can be used for therapeutic interchanging due to the similarity of ingredient profiles in the cluster. Also, members of different clusters can be candidates for new drug combinations as they are expected to affect distinctive biological pathways. Similarly, the cluster of active ingredients in ISN can be used to predict the mechanism of action of newly discovered or synthesized compounds based on TCM classifications. In other words, a functionally-unknown molecule with high structural similarity to any of active ingredient clusters indicate the analogous TCM properties and implications. Therefore, any follow-up experimental analysis can be prioritized to disclose therapeutic hits of the new molecules based on known properties and implications of the corresponding cluster. Also, the priority of active ingredients for treatment can be redefined in each cluster independently using availability, and the relevant protein targets characterizations (code and data set for this case study can be found in **Supplementary File 1**).

**Figure 4:**
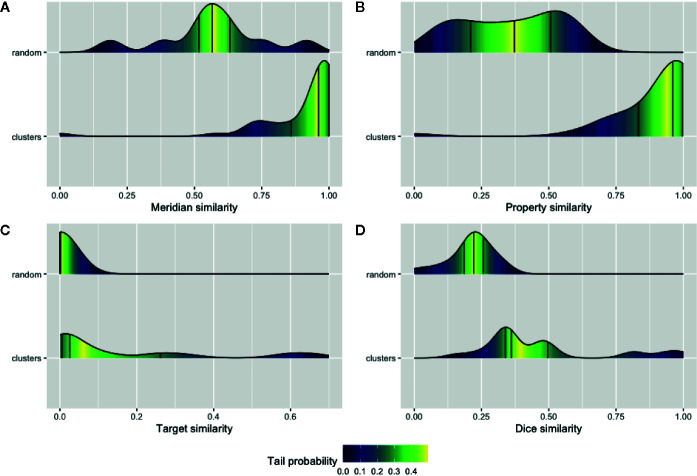
Validation of the network communities in TCM herbs. The distribution of Meridian similarity **(A)** and Property similarity **(B)** within the communities of NSN (natural product similarity network) compared to random groups; The distribution of drug target similarity **(C**, **D)** the Dice index similarity within the communities of ISN (ingredient similarity network) compared to random groups. Lines indicate the quartiles values and gradient color represent the probability of values within the empirical cumulative density function for the distributions.

We sought to validate our prediction about the herb and ingredient communities, i.e., whether the herbs or active ingredients that are clustered in the same community tend to share similar features. Four types of features for the natural products and their ingredients including meridians and properties were extracted from TCMID. Furthermore, the SMILE strings of these ingredients along with the identified or predicted protein targets of them were extracted using PubChem (Kim et al., 2018) and STITCH databases (Szklarczyk et al., 2016). Then, the average of pairwise intersection of meridian and property profiles was computed separately for each cluster in NSN. Likewise, the average similarity of SMILE string using the Dice index and the pairwise intersection of their protein targets in each cluster of ISN were also calculated. We showed in **Figure 4** the average similarity of all the 42 and 24 communities in NSN (**Figures 4A, B**) and ISN (**Figures 4C, D**), as compared to that of random clustering from 100 simulations. We found that the similarity of natural products or active ingredients within a cluster is significantly higher than that for the random clustering. For example, the median of meridian-based similarity of random grouping is 0.56, while the median similarity of the 42 clusters found in NSN is 0.96 (p-value = 1.99e-05, Wilcoxon test). Similarly, in ISN, the median of the Dice similarity of SMILE strings in random groups is 0.22, while the median of the 24 clusters in ISN is 0.36 (p-value = 5.84e-05, Wilcoxon test). Our findings suggested that the clusters of ISN and NSN consist of similar ingredients or natural products, and thus validating the feasibility of bipartite modeling in analyzing TCM data.

Interestingly, these network analyses also suggested a molecular basis of TCM classifications, which originated from the physical features of natural products or empirical knowledge about the disease indications. Although the chemical and molecular characteristics of the natural products, i.e., chemical structures and protein targets have only been available recently, the TCM classification according to meridians were indeed associated with them. The same observation was found for the property classification in TCM in our findings, as the natural products in a given cluster based on ingredient profiles are associated with their property profiles. On the other hand, our approach promises to bridge a gap between pharmaceutical chemistry and traditional pharmacology in TCM. For example, we can use attributes of active ingredient profile of natural products as a rich training set, and newly discovered, or synthesized molecules can be characterized accordingly as a test set. To summarize, this bipartite network analysis provides novel insights for the understanding of molecular evidence of traditional classification in TCM. Using the bipartite network modeling, we may integrate phenotypes of different types, i.e., signs and symptoms, with the chemical knowledge of drug molecules in order to provide a formal framework for phenotype-based drug discovery in TCM.

## Summary and Outlook

Nowadays, TCM, along with the other traditional medical schools, was modernized and expanded by the molecular shreds of evidence provided by experimental biology (Xue et al., 2013). These experiments usually are started by extraction and fractionation techniques such as chromatography, and followed by identification methods such as mass spectrometry to determine a comprehensive profile of ingredients within the natural products (Jafari et al., 2016; Kabiri et al., 2017). The challenging part of this experimental design is identifying the ingredients responsible for the bioactivities of natural products. To further explore the drug-target interactions of these ingredients, high-throughput omics is now a preferable approach to study the effects on gene expression and protein activity. Through these experimental techniques, large volumes of molecular features related to disease indications can be disclosed, including antimicrobial, antiviral, antioxidant, anti-inflammatory, and neurological activities (Neghabi-Hajiagha et al., 2016; Pourramezan et al., 2018). In the next step, we always face the challenge of applying appropriate data integration methods to associate these molecular features with the phytochemical and pharmacological properties of the TCM ingredients.

A remarkable portion of biological studies deal with generating, organizing, and retrieving patient data, which is usually large scale and noisy. Data mining algorithms such as clustering and classification are being applied. Furthermore, the integration of heterogeneous biological data is imperative. Rigorous and efficient analysis tools are required for the integration of different data characteristics and formats as standard statistical inference techniques may be limited (Lee, 2011; Eric, 2014). Harnessing the network modeling in computational biology becomes a feasible strategy for data integration to navigate the complex space of biological systems (Barabási et al., 2011). Here, we highlighted the application of the multipartite network reconstruction for data integration in biomedical researches, particularly in traditional medicine. More specifically, we demonstrated how we can combine current chemical knowledge of ingredients and TCM classifications of natural products to bridge the gap between traditional and modern medicine. We provided a case study to show its potentials for uncovering TCM concepts and discovering potential treatments.

Although network science, and more precisely, network medicine is on its developing stage, using multipartite network modeling may provide more rational on the therapies in traditional medicine. Generally in traditional medicine, much efforts are spent on collecting the symptoms of patients, while in modern medicine, biochemical profiles and image data are more relied on. Providing a framework for integrating all these data using multipartite network model shall facilitate the interchange of knowledge from traditional and modern medicine. Reconstructing multipartite networks is a convenient way to characterize patient similarity, which serves the basis for further explorations on their diseases mechanisms (Pai and Bader, 2018). Depending on the nature of the node sets, a multipartite network can be utilized to investigate complex interactions, that might be critical for understanding diseases with high-level patient heterogeneity such as cancer (Yaffe, 2019). Considering all available data from cellular behaviors to patient responses using multipartite network modeling can play a significant role in the integration of these heterogeneous datasets, a successful application of which may make precision medicine a reality ultimately.

## Author Contributions

Study design: MJ, YW, JT. Data analysis: YW, MJ. Writing: MJ, YW, AA, JT.

## Funding

The work has been supported by the China Scholarship Council (No. 201706740080) and the Finland EDUFI Fellowship (No. TM-18-10928).

## Conflict of Interest

The authors declare that the research was conducted in the absence of any commercial or financial relationships that could be construed as a potential conflict of interest.

## Supplementary Material

Supplementary File 1 R code and dataset for section “Network analysis rationalizes TCM classifications: a case study”.

Supplementary File 2 Modularity index of the clustering algorithms used for the present case study.
